# Onicomadesis como manifestación secundaria de la enfermedad boca-mano-pie

**DOI:** 10.7705/biomedica.7171

**Published:** 2025-05-30

**Authors:** Rodolfo Arredondo-Nontol, Miriam Arredondo-Nontol, Luis Castillo-Peña, Edward Andrés Vertiz, Gaby Lourdes Gómez, Narcisa Reto

**Affiliations:** 1 Departamento Académico de Medicina Humana, Universidad Nacional de Tumbes, Tumbes, Perú Universidad Nacional de Tumbes Departamento Académico de Medicina Humana, Universidad Nacional de Tumbes Tumbes Peru; 2 Unidad Generadora de Evidencias y Vigilancia Epidemiológica - Scientia Clinical and Epidemiological Research Institute, Trujillo, Perú Unidad Generadora de Evidencias y Vigilancia Epidemiológica Scientia Clinical and Epidemiological Research Institute Trujillo Perú; 3 Servicio de Pediatría, Hospital “Carlos Alberto Cortez Jiménez”, EsSalud, Tumbes, Perú Servicio de Pediatría Hospital “Carlos Alberto Cortez Jiménez”, EsSalud Tumbes Perú; 4 Escuela de Medicina Humana, Universidad César Vallejo, Piura, Perú Universidad César Vallejo Escuela de Medicina Humana Universidad César Vallejo Piura Peru

**Keywords:** enfermedades de la uña, enfermedad de boca, mano y pie, enterovirus, reporte de casos., Nail diseases, hand, foot, and mouth disease, enterovirus, case reports.

## Abstract

Se trata de una niña de cinco años, previamente sana, que presentó la enfermedad boca- mano-pie. Un mes después, presentó onicomadesis, es decir, la elevación y posterior separación del lecho ungueal en la región proximal de las uñas del segundo y el tercer dedo de ambas manos, indolora y sin sangrado; posteriormente, se comprometieron todos los dedos de los pies. El cuadro clínico se resolvió espontáneamente durante los tres meses siguientes, con crecimiento de uñas nuevas sanas.

En Latinoamérica, la onicomadesis es una complicación poco descrita de la enfermedad boca-mano-pie y se asocia con formas atípicas de dicha condición, lo cual denota una alteración de la respuesta del huésped a infecciones comunes por el virus Coxsackie. El reporte de este tipo de casos es importante para conocer variaciones de naturaleza benigna y de resolución espontánea, evitar diagnósticos erróneos e informar adecuadamente a los padres.

## Abstract (en)

This case is about a previously healthy five-year-old girl who presented with hand, foot, and mouth disease, and subsequently developed onychomadesis one month later, with detachment of the nail plates on both fingers and toes. However, the condition resolved spontaneously.

After hand, foot, and mouth disease, she exhibited nail bed elevation at the proximal region of the nails on the second and third fingers of both hands. This elevation was painless, without bleeding, and later involved all toes. The condition resolved spontaneously over the following three months, with new, healthy nail growth.

In Latin America, onychomadesis is a rare complication of hand, foot, and mouth disease in our region and is associated with atypical forms of the disease, indicating altered host response to common infections with coxsackie viruses. Reporting such cases is essential to understand these benign and self-limited variations, prevent misdiagnoses, and inform parents appropriately.

La onicomadesis es la separación total o parcial de la lámina ungular en la matriz ungular y puede afectar las uñas de los dedos de las manos o de los pies. Cuando la noxa es intensa y prolongada -e inhibe la matriz ungueal durante dos o más semanas- puede dar origen a una depresión transversal, lo que ocasiona la división de la placa ungular y causa la onicomadesis ^1^^,^^2^.

Las causas descritas relacionadas con la onicomadesis son diversas, generalmente se asocia con enfermedades crónicas autoinmunitarias y debilitantes, como el pénfigo vulgar; a enfermedades de curso agudo y sistémicas, como la enfermedad de Kawasaki, el síndrome de Stevens- Jhonson y la enfermedad de Guillain-Barré; y también, al consumo de fármacos citotóxicos, retinoides y antiepilépticos ^1^. En general, se asume que podría relacionarse con microtraumas y, cuando no se encuentra una causa evidente, con un fenómeno idiopático ^3^. Diversos microrganismos se han relacionado con la onicomadesis, tales como hongos e infecciones por varicela, bacterias causantes de escarlatina y el virus Coxsackie causante de la enfermedad boca-mano-pie ^4^. Esta última es una de las causas poco descritas, aunque la frecuencia de aparición de onicomadesis -especialmente en niños- se ha ido incrementado en los últimos años debido a la emergencia de nuevas cepas virales asociadas con formas atípicas de la enfermedad boca-mano-pie ^5^.

La enfermedad boca-mano-pie es causada por un enterovirus de la familia picornavirus y enterovirus no poliomielíticos, como virus coxsackie A5, A6, A7, A9, A16, B1, B2, B3 y B4, y enterovirus A71. De estos, el enterovirus A71 y el coxsackievirus A16 son los más frecuentes y se han descrito en brotes de Asia y Europa ^6^^,^^7^. La forma típica de la enfermedad se caracteriza por un cuadro febril agudo y lesiones vesiculares no pruriginosas localizadas en la región oral y perioral, las manos y los pies. Usualmente, se resuelve entre seis y diez días, pero, en algunos casos, la enfermedad puede seguir un curso atípico con fiebre prolongada y lesiones extensas pruriginosas y dolorosas en más de cinco partes del cuerpo (nalgas, tronco, codos, orejas y área perioral); esta forma atípica es causada principalmente por el virus coxsackie A6, el cual se ha asociado con la presencia de onicomadesis tras la fase aguda de estas formas atípicas ^8^^,^^9^.

La complicación más común de la enfermedad boca-mano-pie es la meningitis aséptica, que raramente amenaza la vida ^10^. Las infecciones por enterovirus A71 pueden confluir en encefalitis, encefalomielitis, síndrome similar a la poliomielitis, miocarditis, edema, hemorragia pulmonar y muerte ^11^. Los mecanismos etiopatogénicos de la onicomadesis y su relación con la infección viral causante de la enfermedad boca-mano-pie, aún no se entienden bien. Se han postulado desde un daño directo a la matriz ungueal ocasionado por el virus, evidenciado por el aislamiento del virus en fragmentos ungulares, con alteración de la regeneración de la uña; hasta un proceso inflamatorio del tejido periungueal que detendría el crecimiento ungueal y causaría su posterior desprendimiento ^5^^,^^12^.

Los casos de onicomadesis asociados con la enfermedad boca-mano-pie descritos a nivel mundial, proceden principalmente de Europa y Asia, mientras que los reportes de Sudamérica son escasos ^13^. Lo anterior se debe a que, en muchos países latinoamericanos, la enfermedad producida por el virus coxsackie no es de notificación obligatoria y los datos epidemiológicos son limitados debido a la ausencia de vigilancia sistemática de laboratorio ^8^.

Este es un reporte del caso de una niña de cinco años que desarrolló onicomadesis después de un episodio de enfermedad boca-mano-pie. Esta situación causó mucha preocupación a sus padres debido a la naturaleza tardía de los síntomas y al compromiso extenso de la pérdida de uñas.

Se pretende recordar la historia natural de la enfermedad y su conocimiento actual, ya que muchos médicos no están familiarizados con esta forma de presentación y podrían incurrir en un diagnóstico y un manejo erróneos.

## Descripción del caso

Se presenta el caso de una niña de cinco años, producto de un parto por cesárea debido a desproporción cefalopélvica, con peso al nacer de 3.100 g y llanto inmediato; su madre, de 38 años, fue adecuadamente controlada durante la gestación. La infante recibió lactancia mixta, tuvo un desarrollo psicomotor adecuado, inmunizaciones completas para su edad y enfermedades sin importancia.

Dos meses antes de la consulta, dos miembros de la familia fueron afectados por COVID-19: el abuelo materno y la hermana mayor, de nueve años.

El cuadro clínico se inició con un episodio de fiebre (38,6 °C), malestar general y lesiones ulceradas en la faringe posterior. Inicialmente, recibió amoxicilina más ácido clavulánico e ibuprofeno. Sin embargo, un día después, presentó lesiones papulares en la cara, el mentón y el tercer dedo de la mano derecha con prurito moderado. Luego, las lesiones se diseminaron a la región glútea y muchas adquirieron un aspecto pápulo-vesicular con contenido traslúcido. Por las características de estas lesiones, se le diagnosticó enfermedad de boca-mano-pie y se le recomendó no asistir a la escuela.

En los siguientes siete días, las lesiones se extendieron a ambas manos con las mismas características clínicas. La madre reportó que las lesiones de la orofaringe persistieron por cinco días y dificultaban la ingestión de alimentos sólidos. Las lesiones de la cara permanecieron por una semana y, las del área glútea, hasta por dos semanas, periodo durante el cual la paciente logró recuperarse y retornó al colegio. Tras la recuperación, la madre no observó descamación superficial en las zonas afectadas, ni tampoco ninguna lesión inflamatoria alrededor de las uñas de las manos o los pies.

Un mes después de iniciarse la enfermedad boca-mano-pie, la madre notó una elevación del lecho ungueal en la zona proximal de las uñas del segundo y tercer dedos de ambas manos, por lo que acudió a consulta médica. La elevación del lecho ungueal no causaba dolor, ni sangrado. La madre negó antecedentes de traumatismos o contacto con detergentes o sustancias corrosivas.

**Figura 1:**
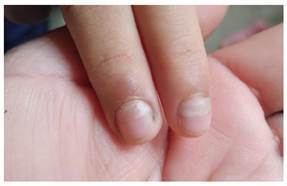
Onicomadesis del segundo y el tercer dedo de la mano de una niña, posterior a la enfermedad boca-mano-pie

En el examen físico, los signos vitales fueron normales y la paciente se encontró eutrófica, despierta, con adecuada hidratación, lúcida y colaboradora. Tenía la piel tibia (normotérmica) sin lesiones visibles. La placa ungueal de la región proximal de las uñas del segundo y tercer dedo de las manos estaba desprendida, sin evidencia de signos traumáticos (figura 1). Las uñas de los pies no presentaban alteraciones. No se evidenciaron anomalías estructurales o deformidades en los dedos de las manos o los pies.

Se realizó un examen directo para la detección de hifas de hongos, el cual resultó negativo. El padre no autorizó los estudios de bioquímica ni hematología sanguínea.

**Figura 2:**
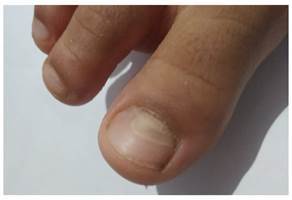
Onicomadesis de las uñas de los pies de una niña, posterior a la enfermedad boca-mano-pie

**Figura 3.**
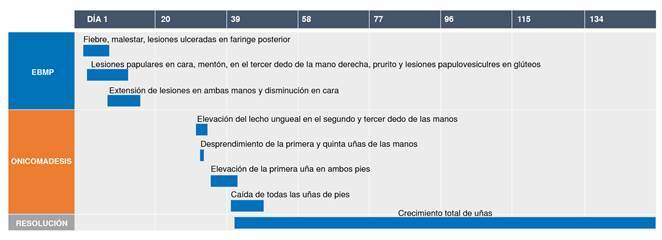
Línea del tiempo de la paciente con onicomadesis posterior a la enfermedad boca-mano-pie

Se hizo un seguimiento telefónico de la evolución de la caída de las uñas. La madre informó que, al día siguiente de la consulta, la menor presentó desprendimiento del lecho ungueal en las uñas del primer y quinto dedos de ambas manos; dos días después, notó el levantamiento de la primera uña de ambos pies y, en los cinco días siguientes, el levantamiento se extendió progresivamente a todas las uñas de los pies (figura 2). El consiguiente desprendimiento de las uñas ocurrió en el mismo orden de afectación y fue indoloro. El número total de uñas afectadas fue de 17 (siete en las manos y diez en los pies). Se estableció el diagnosticó de onicomadesis. Todas las uñas afectadas fueron reemplazadas por nuevas durante los tres meses siguientes. Las nuevas uñas no mostraron ninguna alteración en cuanto a transparencia o dureza (figura 3).

EBMP: enfermedad boca-mano-pie

### Consideraciones éticas

Se obtuvo el consentimiento informado de los padres de la paciente para la publicación del presente caso, así como de las imágenes. Los autores se aseguraron de no incluir ningún dato personal. La redacción y revisión del caso se llevó a cabo con el visto bueno del Comité de Ética del Hospital Carlos Alberto Cortez Jiménez, donde se atendió a la paciente, como certifica la Constancia No. 006/2023 CEI-HCACJ-ESSALUDTUMBES.

## Discusión

Este es un caso de onicomadesis en una niña pequeña, por lo demás sana, sin antecedentes patológicos de importancia, y que se asoció con una infección previa diagnosticada clínicamente como la enfermedad boca-mano- pie. Al respecto, existen reportes similares de episodios de onicomadesis asociados con esta enfermedad, en los cuales el coxsackievirus A6 fue el agente aislado con mayor frecuencia ^7^.

La patogenia de la onicomadesis aún es motivo de investigación. Se han planteado varios factores, como la inhibición temporal de la proliferación de la matriz ungueal, la propagación directa de la inflamación a partir de las lesiones cutáneas alrededor de las uñas, la afectación específica de la matriz ungueal por virus Coxsackie y el compromiso sistémico grave generado por la enfermedad boca-mano-pie de niños pequeños ^5^.

Es probable que el virus Coxsackie 6 haya evolucionado cambiando sus características y presente nuevas interacciones con el huésped, lo que puede producir manifestaciones clínicas diferentes, no solo en la etapa aguda, sino también, en la etapa posterior. La onicomadesis podría ser una manifestación de ello ^5^.

La mayoría de los reportes de onicomadesis se originan en Europa y Asia, donde son frecuentes los brotes de coxsackievirus. Sin embargo, en Latinoamérica la información es limitada, probablemente debido a la poca capacidad de aislamiento viral en estos países ^13^. La onicomadesis se ha asociado con otros signos que implican daño de la matriz ungular, como las líneas de Beau, pero esto no se observó en el presente caso ^9^. La afectación ungueal característica se origina inicialmente como una grieta en la base de la uña, la cual se fractura y se desprende de la placa, tal como sucedió en este caso ^7^.

Se ha informado un tiempo de tres a ocho semanas entre la infección por coxsackievirus y la aparición de la onicomadesis. En el presente caso, las manifestaciones se dieron alrededor de las cuatro semanas después, periodo dentro del rango promedio reportado ^14^. El número de uñas afectadas oscila entre 1 y 9, con un promedio de 2. En este caso, llama la atención el gran compromiso ungueal que presentó la niña, con afectación de 17 uñas: siete de las manos y diez de los pies ^9^.

Entre los agentes implicados en el desarrollo de la enfermedad boca- mano-pie, el coxsackievirus A6 es el más frecuente. Este se ha asociado con episodios más graves y de mayor compromiso, con manifestaciones atípicas que comprenden lesiones polimorfas y extensas en la cara, los pies, los muslos y la región glútea, como en este caso ^8^. Con base en estos datos clínicos, es muy probable que, dada la afectación observada en la paciente -lesiones pruriginosas extendidas más allá de las manos y los pies, y el desarrollo de onicomadesis-, se sospeche la posibilidad de una infección por coxsackievirus A6. Por consiguiente, es probable que este agente circule en la región ocasionando formas atípicas y graves, sin diagnóstico claro. En Latinoamérica, a diferencia de Asia, la infección por coxsackievirus no es de notificación obligatoria y existen limitaciones para el aislamiento del virus ^13^.

La presentación atípica de la onicomadesis dificulta su diagnóstico y, por ser fácilmente transmitida, se incrementa el contagio sobre todo en las escuelas; tampoco existen vacunas para prevenir la infección por coxsackievirus ^6^. El personal de salud también podría estar haciendo diagnósticos inadecuados debido al desconocimiento de esta secuela atípica de la enfermedad boca-mano-pie ^15^.

Este informe de caso tiene como objetivo ilustrar una complicación atípica de la enfermedad boca-mano-pie, cuya forma de presentación, además de causar gran preocupación en los padres, origina diagnósticos erróneos por parte de los profesionales de la salud. El reporte de estos casos atípicos ayuda a comprender mejor la onicomadesis y, también, a tranquilizar a la familia sobre su curso clínico, que es autolimitado.

## References

[1] Lee DK, Lipner SR. (2022). Optimal diagnosis and management of common nail disorders. Ann Med.

[2] Alghamdi A, Mazraani N, Alghamdi Y, Albugami SM. (2022). Onychomadesis and Beau’s line following hand-foot-and-mouth disease in a seven-year-old male. Cureus.

[3] Smith RJ, Rubin AI. (2020). Pediatric nail disorders: A review. Curr Opin Pediatr.

[4] Eguía Angeles HA, Sotelo García CO, Vadæle J, Rasmussen H, Eguía Angeles EA. (2018). Onychomadesis in an immunocompetent adult patient. Semergen.

[5] Chiu HH, Liu MT, Chung WH, Ko YS, Lu CF, Lan CC (2019). The mechanism of onychomadesis (Nail shedding) and Beau’s lines following hand-foot-mouth disease. Viruses.

[B6] Sharma A, Mahajan V, Mehta K, Chauhan P, Manvi S, Chauhan A. (2022). Hand, foot and mouth disease: A single centre retrospective study of 403 new cases and brief review of relevant Indian literature to understand clinical, epidemiological, and virological attributes of a longlasting Indian epidemic. Indian Dermatol Online J.

[B7] Zhao TS, Du J, Sun DP, Zhu QR, Chen LY, Ye C (2019). A review and meta-analysis of the epidemiology and clinical presentation of coxsackievirus A6 causing hand-foot-mouth disease in China and global implications. Rev Med Virol.

[B8] Justino MCA, da S. Mesquita D, Souza MF, Farias FP, Jainara JC,, Ferreira JL (2020). Atypical hand-foot-mouth disease in Belém, Amazon region, northern Brazil, with detection of coxsackievirus A6. J Clin Virol.

[B9] Li D, Wu Y, Xing X, Huang J, Mao A, Liu T (2019). Onychomadesis and potential association with HFMD outbreak in a kindergarten in Hubei province, China, 2017. BMC Infect Dis.

[B10] Saguil A, Kane SF, Lauters R, Mercado MG. (2019). Hand-foot-and-mouth disease: Rapid evidence review. Am Fam Physician.

[11] Wong SS, Yip CC, Lau SK, Yuen KY. (2010). Human enterovirus 71 and hand, foot, and mouth disease. Epidemiol Infect.

[12] Cui Y, Shi Q, Song P, Tong J, Cheng Z, Zhang H (2024). Coxsackievirus A10 impairs nail regeneration and induces onychomadesis by mimicking DKK1 to attenuate Wnt signaling. J Exp Med.

[13] Giordano MC, de la Fuente A, Lorca MB, Kramer D. (2018). Onicomadesis secundaria a enfermedad pie-mano-boca: una manifestación frecuente y motivo de preocupación de los padres. Rev Chil Pediatr.

[14] Li XW, Ni X, Qian SY, Wang Q, Jiang RM, Xu WB (2018). Chinese guidelines for the diagnosis and treatment of hand, foot, and mouth disease (2018 edition). World J Pediatr.

[15] Cheng FF, Zhang BB, Cao ML, Zhang Q, Chen QH, Hui ZF (2022). Clinical characteristics of 68 children with atypical hand, foot, and mouth disease caused by coxsackievirus A6: A single-center retrospective analysis. Transl Pediatr.

